# Macrophages facilitate the excystation and differentiation of *Toxoplasma gondii* sporozoites into tachyzoites following oocyst internalisation

**DOI:** 10.1038/srep33654

**Published:** 2016-09-19

**Authors:** Wesley Freppel, Pierre-Henri Puech, David J. P. Ferguson, Nadine Azas, Jitender P. Dubey, Aurélien Dumètre

**Affiliations:** 1 Aix Marseille Université, IP-TPT UMR MD 3, Marseille, F-13385, France; 2 Aix Marseille Université, LAI UM 61, Marseille, F-13288, France; 3 Inserm, UMR_S 1067, Marseille, F-13288, France; 4 CNRS, UMR 7333, Marseille, F-13288, France; 5 Nuffield Department of Clinical Laboratory Science, University of Oxford, John Radcliffe Hospital, Oxford, OX3 9DU, United Kingdom; 6 United States Department of Agriculture, Agricultural Research Service, Beltsville Agricultural research Center, Animal Parasitic Diseases Laboratory, Building 1001, Beltsville, MD 20705-2350, USA

## Abstract

*Toxoplasma gondii* is a common parasite of humans and animals, which is transmitted via oocysts in cat faeces or tissue cysts in contaminated meat. The robust oocyst and sporocyst walls protect the infective sporozoites from deleterious external attacks including disinfectants. Upon oocyst acquisition, these walls lose their integrity to let the sporozoites excyst and invade host cells following a process that remains poorly understood. Given the resistance of the oocyst wall to digestive enzymes and the ability of oocysts to cause parenteral infections, the present study investigated the possible contribution of macrophages in supporting sporozoite excystation following oocyst internalisation. By using single cell micromanipulations, real-time and time-point imaging techniques, we demonstrated that RAW macrophages could interact rapidly with oocysts and engulfed them by remodelling of their actin cytoskeleton. Internalised oocysts were associated to macrophage acidic compartments and showed evidences of wall disruption. Sporozoites were observed in macrophages containing oocyst remnants or in new macrophages, giving rise to dividing tachyzoites. All together, these results highlight an unexpected role of phagocytic cells in processing *T. gondii* oocysts, in line with non-classical routes of infection, and open new perspectives to identify chemical factors that lead to oocyst wall disruption under physiological conditions.

*Toxoplasma gondii* is a common coccidian parasite causing both acute and chronic infections in humans and animals^1^. It is estimated that approximately a third of the global human population is infected by the parasite. Human infections can arise from ingesting either sporulated oocysts from food or water contaminated with cat faeces or tissue cysts present in the meat from infected animals. The relative importance of either route of infection is still unclear and depends on the local environment and habits of the citizens. It has been estimated that approximately half of infections are due to ingestion of environmentally resistant oocysts with water and food^2^. Infective *T. gondii* oocysts are ovoid and measure 11 × 13 μm in size and contain two sporocysts (6 × 8 μm), each of them hosting four haploid sporozoites (2 × 6–8 μm)^3^. Both oocyst and sporocyst walls are bilayered and appear to be made of similar structural polymeric molecules, mainly cross-linked tyrosine-rich proteins^4^,^5^,^6^,^7^. These walls are believed to be mainly passive materials that protect the sporozoites from deleterious physical and chemical attacks the oocyst may experience outside/inside the host^3^,^8^. The oocyst wall acts as a primary barrier to external injuries and resists chemical disinfectants. For instance, exposing oocysts to household bleach solution causes the degradation of the outer layer of the oocyst wall but has little effect on the structure and mechanics of the inner layer^8^ nor on the viability of the sporozoites. The sporocyst bilayered wall appears to represent a second level of protection for the sporozoites^3^,^9^,^10^, though its capacity to resist physical and chemical attacks is relatively unknown. The unique structure of the sporocyst inner wall layer, which consists of four curved plates tightly joined together by suture lines, would provide mechanical resistance^10^. The sporozoite is similar in structure to the other infective stages of the parasite (tachyzoite and bradyzoite) but with slight differences in the numbers of apical organelles and the presence of polysaccharide-containing granules^11^,^12^.

Upon ingestion, the *T. gondii* oocyst enters the host digestive tract and is thought to result in sporozoites reaching the small intestinal epithelium within hours^13^,^14^,^15^. This requires the rupturing of both the oocyst and sporocyst walls to allow the sporozoites to escape and invade the host cells. This essential process, called excystation, is proposed to involve at least one physical stimulus of unknown nature in conjunction with the action of digestive agents, pH, and dissociated carbon dioxide that the oocyst likely encounters in the host digestive tract^16^,^17^,^18^. Whether the intestinal cells contribute to the opening of the oocyst walls and favour sporozoite excystation is unknown. However, there is an interesting reported and not yet fully understood anomaly in that *T. gondii* oocysts are also infective following parenteral inoculation (e.g. through subcutaneous injection in mice); the infectivity of oocysts by the subcutaneous route paralleled the oral route^19^,^20^. The phenomenon of infection from parenteral inoculation has also been reported for the closely coccidian parasites belonging to the genus *Eimeria*^21^,^22^. This unusual behaviour among coccidian parasites suggests that contact between the host digestive microenvironment and the oocyst is not the only route for opening the robust walls of the *T. gondii* oocyst. This fact raises question about the possible contribution of host cells, in particular of which cell type, in promoting sporozoite excystation at extra-intestinal sites.

The capacity of naïve macrophages to ingest large particles (10–20 μm in diameter)^23^ besides their inability to eliminate the sporozoite and tachyzoite forms of *T. gondii*^11^,^13^,^14^,^24^,^25^ prompted us to investigate whether the macrophages were able to internalise *T. gondii* oocysts, open their wall, hence allowing the sporozoites to excyst prior to their differentiation into tachyzoites. To address this question, we investigated the direct interactions between *T. gondii* oocysts and RAW murine macrophages at different time points by using single cell and parasite micropipette micromanipulations and a combination of real-time and time-point imaging techniques. Our results demonstrate that RAW macrophages are able to internalise efficiently *T. gondii* oocysts in a time-dependant manner and then to cause the opening of the walls of engulfed oocysts in co-culture conditions. We present data highlighting an unexpected role for the macrophage in facilitating the excystation and differentiation of *T. gondii* sporozoites following oocyst internalisation.

## Results and Discussion

### The macrophage can adhere rapidly to oocysts and start their internalisation within minutes following initial contact

**Figure 1:**
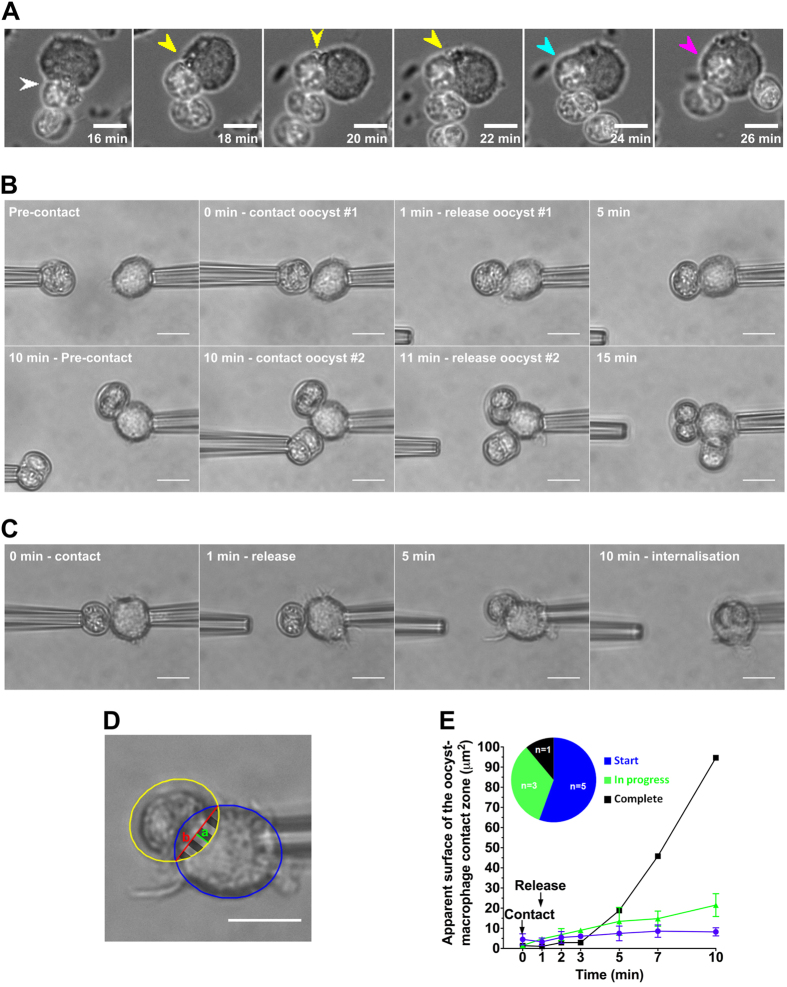
Analysis of initial interactions between RAW macrophages and native *T. gondii* oocysts recorded by time-lapse video microscopy at 37 °C (**A**) and by using micropipette techniques at room temperature (**B–E**). (**A**) Selected frames recapitulating the different steps of the oocyst internalisation by macrophage cells: oocyst adhesion (white arrowhead), macrophage membrane extension (yellow), closure of the phagocytic cup (blue), and full internalisation (magenta). Here, the process starts at t = 16 min, i.e. 16 min after introducing oocysts in the macrophage cell culture (at this time, after sedimentation, most of oocysts located in the same focal plane as the macrophage cells). Scale bar: 10 μm. (**B**) After one minute of contact, a first oocyst adhered to a macrophage and processed by it (i.e. moved around). A second oocyst was presented to the macrophage at t = 10 min, released at t = 11 min, and was also processed by the macrophage. Scale bar: 10 μm. (**C**) Follow-up of the complete internalisation of one oocyst by a micropipette-held macrophage. Scale bar: 10 μm. (**D**) The positions of the macrophage and the oocyst were recorded by elliptical blue and yellow overlays respectively. The apparent surface of the contact zone (striped area, *A*) was calculated by measuring the respective length of the segments *a* (green) and *b* (red) and then by using the following formulae: 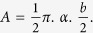 Scale bar: 10 μm. (**E**) Plot of the apparent surface of the oocyst-macrophage contact zone over time.

RAW macrophages were mixed with oocysts in a 1:1 ratio at 37 °C and the subsequent interactions followed live by phase contrast microscopy and recorded using time-lapse microscopy (Fig. 1A). It was observed that the macrophages immediately reacted to the presence of the oocysts (sporulated or unsporulated) by producing pseudopod-like cellular extensions stretching towards the oocysts (Fig. 1A, Supplementary Movie S1). These extensions attached to the oocysts and once attached, oocysts were not released by the macrophage and pulled toward the cell (Movie S1) After attachment, the macrophage cytoplasm surrounded the oocyst in a phagocytic-like manner leading to full internalisation within 10 min (Fig. 1A, Supplementary Movie S2). The macrophages could attach to several oocysts in a sequential manner and repeated phagocytosis resulted in macrophages containing multiple oocysts (Supplementary Movie S3). On occasions, multiple predator cells can appear to compete for a single oocyst with each macrophage pushing forward pseudopodia (Supplementary Movie S4).

To further analyze the initial interaction between macrophages and oocysts, cells and parasites were individually manipulated and presented to each other by using micropipettes (Fig. 1B). Following an initial contact of 1 min during which the two partners were gently pressed together, the oocyst was released from its holding pipette and its possible internalisation by the macrophage monitored over 10 min at room temperature. Within one minute of contact, the oocyst was tightly adherent to the macrophage with none of the oocysts subsequently being released (Fig. 1B,C). Once adherent, there was evidence of progressive internalisation with the macrophage cytoplasm engulfing the oocyst (Fig. 1C–E). However, there were marked differences in the rate of internalisation of the individual oocysts over the 10 min period examined. The all (9 of 9 oocysts examined) remained adherent while a proportion also showed partial (3 of 9) or more rarely full (1 of 9) internalisation of the oocyst (Fig. 1E). To confirm that macrophages can interact with multiple oocysts simultaneously, a second oocyst was presented to a macrophage with an adherent oocyst. It was observed that the macrophage could retain the second oocyst and initiate partial internalisation of both oocysts in the limited time examined (Fig. 1B). Together with time-lapse video microscopy data obtained at physiological temperature (see above), micromanipulation experiments highlighted that unperturbed, native *T. gondii* oocysts remain tightly adherent to the naive macrophage following rapid contact and be processed for engulfment within 10 min even at room temperature. These results are consistent with studies using spherical particles of 10–20 μm size as models and cell-line macrophages^23^ or blood-isolated neutrophils^26^.

### RAW macrophages efficiently internalise native *T. gondii* oocysts in a time dependant manner

**Figure 2:**
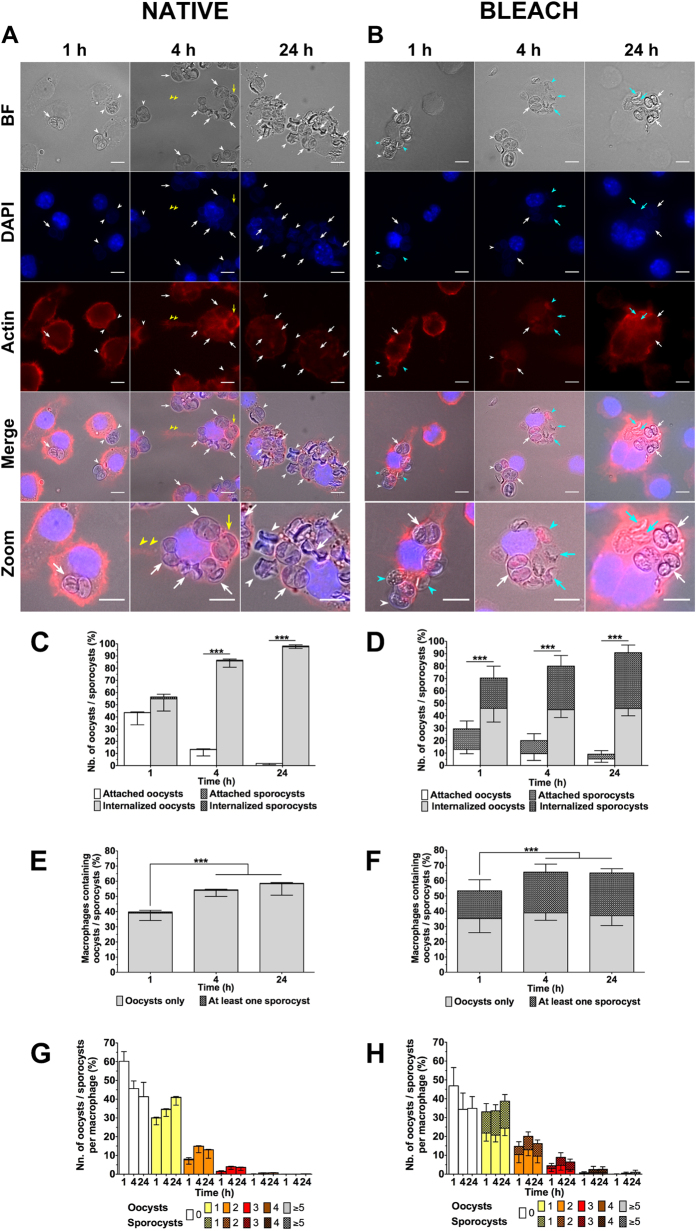
Internalisation of native or bleach-treated oocysts *T. gondii* oocysts by RAW macrophages. (**A,B**) Macrophage cells were incubated with native or bleach-treated oocysts at a ratio of 1:1 for 1, 4 and 24 h at 37 °C and then fixed and stained for macrophage nucleus (blue) and actin cytoskeleton (red) identification. Actin staining allowed to distinguish internalised (white arrow) from attached (white arrowhead) oocysts, in particular freshly internalised ones (yellow arrow), and additionally long pseudopod-like extensions of the macrophage cytoplasm (yellow arrowhead). Light blue arrowheads and arrows denoted sporocysts that adhered to or were internalised by macrophages, respectively. Scale bars: 10 μm. Internalisation assays allowed to calculate (**C,D**) the number of oocysts/sporocysts attached to or internalised by macrophages, (**E,F**) the number of macrophages containing oocysts/sporocysts, and (**G,H**) the number of internalised oocysts/sporocysts per macrophage. ***p < 0.001.

To investigate the ability of RAW macrophages to internalise *T. gondii* oocysts, macrophage cells were incubated at 37 °C in a 1:1 ratio with native oocysts for 1, 4 or 24 h, subsequently fixed and permeabilized, and their actin cytoskeleton stained with phalloidin in order to discriminate attached versus internalised oocysts substructures (i.e. unsporulated or sporulated oocysts or more rarely free sporocysts) (Fig. 2A). It was possible to observe the pseudopod-like cellular extensions attaching to remote oocysts (Fig. 2A, yellow arrowheads) and cytoplasmic processes progressively surrounding the oocysts (phagocytising) (Fig. 2A, yellow arrows), similar to what was observed for short interaction times (Fig. 1A,C). Internalised oocysts appeared to be deformed and showed evidences of wall damages especially at 4 and 24 h (Fig. 2A). The wall of many sporocysts appeared collapsed at the plate junctions and some sporozoites were no longer visible in damaged sporocysts starting from 4 h of incubation (Fig. 2A). To quantify the interactions, the ratio of the number of internalised versus attached oocysts/sporocysts (Fig. 2C), the ratio of number of macrophages containing one or more oocysts/sporocysts to total number of macrophages (Fig. 2E), and the number of internalised oocysts/sporocysts per macrophage (Fig. 2G) were calculated from the time point images. It was observed that there was a significant increase over time in the number of internalised compared to adherent oocysts with 50, 80, and 90% of the oocysts internalised by the macrophages at 1, 4, and 24 h respectively (Fig. 2C). In addition, there was a significant increase in the number of macrophages containing oocysts with at least 55% of macrophages hosting one or more oocysts at 24 h (Fig. 2E). Macrophages typically contained one or two oocysts (Fig. 2G), however a few macrophages with more oocysts (up to 8) were observed at 24 h (Fig. 2A,G).

### Removal of the outer oocyst wall layer did not significantly modify the dynamics of oocyst internalisation

The *T. gondii* oocyst wall is mainly made of structural cysteine and tyrosine-rich proteins^4^,^5^,^6^,^7^,^27^ with little evidence of additional molecules that could mediate interactions with host cells, i.e. PAN-domain-containing proteins in the outer layer^5^ and the macrophage dectin-1 ligand beta-1,3 glucan located in the inner layer^28^. To examine whether modifications of the structure of the oocyst wall affected oocyst adherence to and internalisation by macrophages, oocysts were exposed to a bleach solution in order to strip off the outer oocyst wall layer^8^. The interactions of the macrophages and bleach-treated oocysts were quantified at 1, 4 or 24 h time points using the criteria described above (Fig. 2B). As observed for native oocysts, bleach-treated oocysts examined in macrophages were frequently damaged and their internal content no longer visible (Fig. 2B). The change in adherence and internalisation by macrophages as well as the number of oocyst substructures per macrophage showed similar changes with time as the native oocysts (comparing Fig. 2D,F,H to Fig. 2C,E,G respectively). This behaviour suggests that the molecules exposed at the surface of native or bleach-treated oocysts do not have a significant role in the oocyst-macrophage interaction. However, there was a marked increase in the number of free sporocysts in bleach-treated samples (Fig. 2B,D,F,H). This would be consistent with an increase in the fragility of the wall of some oocysts due to the removal of the outer layer following bleach exposure. The level of internalisation of the sporocysts significantly increased at 1 h in the bleach-treated samples (Fig. 2D). This was possibly related to a more rapid internalisation of the sporocysts because of their smaller size (6 × 8 μm) than intact oocysts (11 × 13 μm)^29^.

### Effects of macrophage actin cytoskeleton inhibitors on macrophage-oocyst interaction

**Figure 3:**
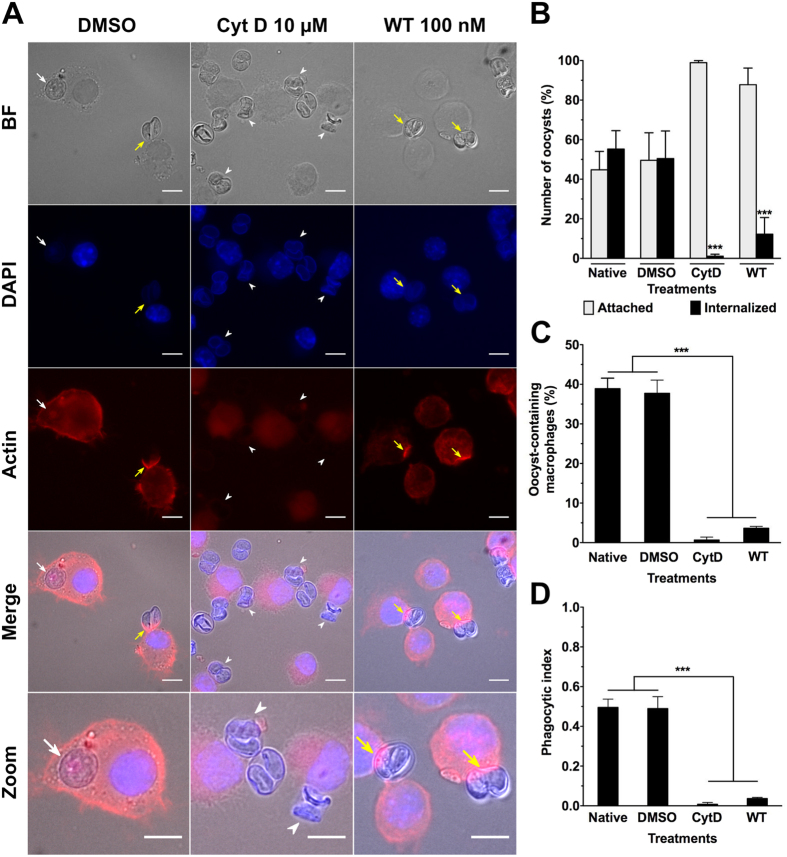
Internalisation of native *T. gondii* oocysts is significantly inhibited following treatment of RAW macrophages with actin polymerization or PI3 kinase inhibitors. (**A**) Representative images of macrophages pretreated for 1 h at 37 °C with either 10 μM actin inhibitor cytochalasinD (CytD) or 100 nM PI3K inhibitor wortmannin (WT) and then incubated for 1 h with native oocysts at 37 °C in presence of each inhibitor. After incubation, cells were fixed and stained for their nucleus and actin cytoskeleton visualization. Untreated and DMSO-treated macrophage cells served as controls. Actin staining allowed identification of internalised (white arrow) from attached (white arrowhead) oocysts, and visualization of actin recruitment at the nascent phagocytic cup (yellow arrow). Scale bars: 10 μm. (**B–D**) Comparative quantitation after treatment with CytD or WT: (**B**) number of oocysts attached to or internalised by macrophages, (**C**) number of macrophages containing oocysts, and (**D**) corresponding phagocytic index. ***p < 0.001.

Rearrangement of the actin cytoskeleton of phagocytic cells typically drives internalisation of foreign particles^30^,^31^. To examine the contribution of the actin network of the macrophage in the uptake of oocysts, the macrophages were treated with cytochalasin D (CytD) (F-actin polymerisation inhibitor) or wortmannin (WT) (phosphoinositide 3-kinase (PI3K) inhibitor). The latter impairs the closure of phagosomes but not the phagocytic cup formation^32^,^33^. In the presence of 10 μM CytD or 100 nM WT, macrophages incubated with native oocysts for 1 h exhibited normal rates of attachment but very little evidence of internalisation of the oocysts compared to untreated control macrophages (Fig. 3A,B). Both the untreated and WT samples showed actin recruitment to the phagocytic cup, but not CytD-treated macrophages (Fig. 3A). These observations are consistent with actin being required to start oocyst engulfment but this process cannot be completed in the presence of WT. As a consequence, the number of macrophages containing oocysts as well as the corresponding phagocytic index was also significantly reduced compared to controls (Fig. 3C,D). These observations were consistent with oocyst internalisation involving active remodelling of the macrophage actin cytoskeleton as a first step^30^.

### Expression and localization of acidic granules upon macrophage-oocyst co-incubation

**Figure 4:**
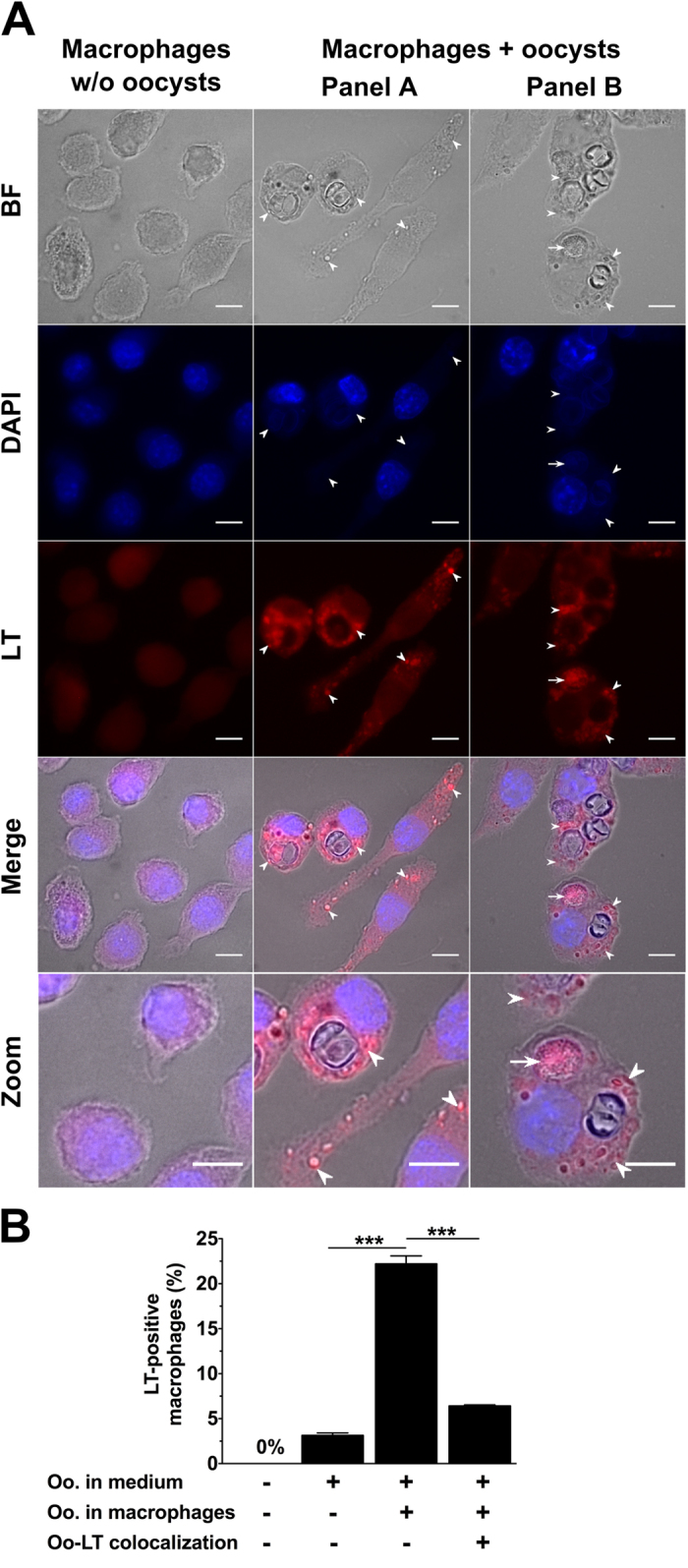
Expression and localization of acidic granules in macrophages following a 24-h co-incubation period with native *T. gondii* oocysts. (**A**) Acidic granules (arrowhead) were tracked by incubating the cells with 100 nM red LysoTracker^®^ DND-99 (LT) for 24 h at 37 °C. After fixation, cells were observed under bright field (BF), UV illumination for oocyst autofluorescence and DAPI staining of the macrophage nucleus, and the appropriate filter set for LT fluorescence. Arrow indicates LT-oocyst colocalization. Macrophages cultured in absence of oocysts served as negative controls. Scale bar: 10 μm. (**B**) The number of LT-positive macrophages were determined over 800 cells counted per experiment. ***p < 0.001 (n = 4).

The possible onset of acidic granules surrounding or co-localizing with oocysts inside the macrophages was investigated by using the red fluorescent LysoTracker dye (Fig. 4A,B). Macrophages that were incubated in the absence of oocysts did not harbour any LysoTracker-positive granules (Fig. 4A). In macrophages exposed to oocysts, the acidic granules were predominately located around the ingested oocysts (Fig. 4A, panels A and B) rather than colocalizing (panel B) with acidic vacuoles (22.2 ± 0.9% vs. 6.4 ± 0.1%, p < 0.001) (Fig. 4B). It was noted that acidic compartments often co-localized with unsporulated rather than sporulated oocysts. Interestingly, LysoTracker-positive granules were also observed, in co-cultures, in several macrophages with no detectable oocyst substructures in their cytoplasm, but located in the close vicinity of oocyst-containing macrophages (Fig. 4A, panel A). This could suggest that incubation with oocysts alone was sufficient to stimulate lysosome formation (Fig. 4A). Opening of the oocyst and sporocyst walls appears in these experiments to occur within macrophages, possibly following exposition of the parasites to the content of the acidic compartments and internal forces. The oocyst wall of *T. gondii* is highly resistant to acid pH, enzymatic degradation and protease activity^18^,^34^. As macrophage phagolysosome contains numerous molecules that can mediate degradation of foreign microorganisms^35^, it remains unclear which chemical factors would lead to wall disruption. Interestingly, it was shown that *T. gondii* oocysts can be ingested by free-living amoeba, which are ancestral phagocytic cells, resist phagocytic lysis, retain their viability and establish infection in mice^36^. Similarly, macrophage cells were reported to be able to engulf and open *Eimeria acervulina* oocysts or sporocysts, and to play a key role in the transport of the subsequent sporozoites from subcutaneous tissues towards the intestine where the parasites multiply^37^. Whether the sporozoites excysted inside or outside the macrophages or other cells were not investigated^37^.

### Evidence for Toxoplasma development after co-culturing

**Figure 5:**
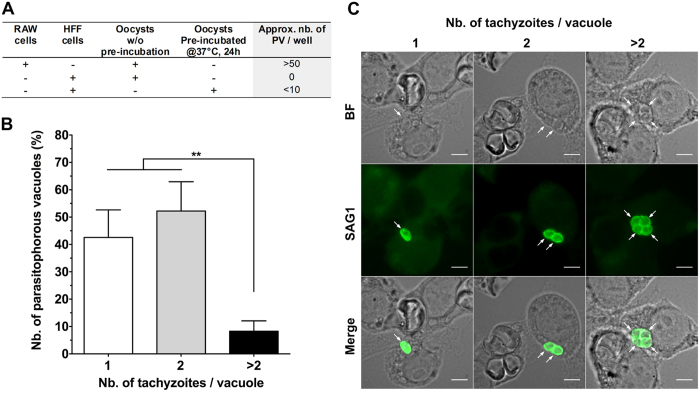
Detection of *T. gondii* tachyzoites in RAW macrophages following a 24-h co-incubation period with native *T. gondii* oocysts. Following oocyst co-incubation, macrophage or HFF cells were fixed and permeabilized with methanol and tachyzoites were detected by using a tachyzoite specific SAG1 polyclonal antibody. (**A**) Semi-quantification of the number of parasitophorous vacuoles (PV) detected in RAW cells or HFF cells from four independent experiments. HFF cells incubated with oocysts, either pre-incubated at 37 °C for 24 h or not, served as controls for possible sporozoite excystation in absence of phagocytosis by RAW cells, as described in the materials and methods section. (**B**) Distribution of the number of tachyzoites detected per PV (over ~50 PV counted) in RAW cells incubated with native oocysts for 24 h **p < 0.01. (**C**) Representative images of tachyzoites identified in RAW cells. Scale bars = 5 μm.

The possible excystation of sporozoites from oocysts within macrophages and their subsequent differentiation into tachyzoites were investigated after 24 h of incubation. Earlier *in vitro* and *in vivo* studies reported that, at this time, most of excysted sporozoites were transformed into tachyzoites in mononuclear, endothelial or fibroblast cell types^13^,^38^,^39^,^40^. As *T. gondii* tachyzoites, but neither sporozoites nor oocyst walls, expressed at their surface the SAG1 protein^40^, macrophage cells were examined for the presence of SAG1 + tachyzoites. Under these experimental conditions, it was possible to identify tachyzoites within the cytoplasm of a few macrophages (>50 tachyzoite-containing vacuoles/well) (Fig. 5A). There was also evidence of proliferation with many vacuoles containing two parasites and a few four or more parasites (Fig. 5B,C). The tachyzoite-containing vacuoles were often observed in macrophages that did not harbour any visible oocyst substructures but in proximity of cells hosting damaged sporulated oocysts (Fig. 5C). To examine the specific role played by macrophages, oocysts were incubated with epithelial cells (HFF) for 24 h at 37 °C and examined for the presence of developing tachyzoites : no tachyzoite development was observed (Fig. 5A). However, to verify if pre-incubation of oocysts at 37 °C in a cell free environment can stimulate excystation, oocysts were pre-treated at 37 °C for 24 h before incubating with HFF cells. Surprisingly, a few tachyzoite-containing vacuoles (i.e. <10 vacuoles containing tachyzoites each/well) were detected in HFF cells (Fig. 5A). It is possible that increasing the temperature to 37 °C alone could have stimulated a few oocysts to excyst, but this appeared to occur at a much lower rate than following oocyst/macrophage interactions. In a separate set of experiments, we then compared tachyzoite development in macrophages challenged for 24 hr with intact oocysts or free sporozoites obtained following oocyst sonication and exposure of the released sporocysts to bile salt solution. Tachyzoites developed in both cases with no or very little evidence of sporozoites persisting in co-cultures (Supplementary Fig. S1). The percentage of macrophages containing tachyzoites was higher following macrophage challenge with bile-excysted sporozoites (Supplementary Fig. S1A). This could suggest that motile sporozoites infected cells they encountered while macrophages needed to move to internalise static oocysts. Altogether, these results confirm the important role of the macrophage in oocyst excystation in absence of a forced unnatural physical stimulus (sonication) and digestive components such as bile extract.

### Ultrastructural observations of macrophage-oocyst interactions

**Figure 6:**
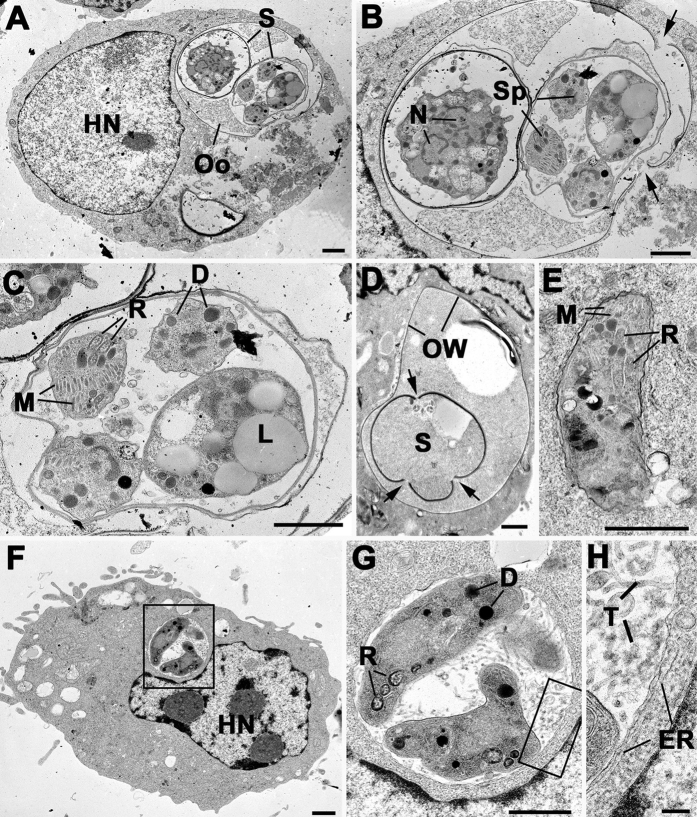
Ultrastructural observations of macrophage-oocyst interactions. (**A**) Low power image showing a macrophage containing an intact oocyst (Oo) and sporocysts (S). HN – host cell nucleus Bar is 1 μm. (**B**) Enlargement of the oocyst showing one sporulated sporocyst with sporozoite (Sp) and an unsporulated sporocyst with the cytoplasmic mass containing multiple nuclei (N). Note the breaks in the oocyst wall (arrows). Bar is 1 μm. (**C**) Detail of the sporocyst showing the sporozoites containing the characteristic apical organelles of rhoptries (R), micronemes (M) and dense granules (**D**) and the residual body containing lipid droplets (L). Bar is 1 μm. (**D**) Part of a macrophage containing an oocyst in which there is an “empty” sporocyst showing breaks in the sporocyst wall (arrows). Bar is 1 μm. (**E**) Sporozoite exhibiting rhoptries (R) and micronemes (M) located within a tight fitting parasitophorous vacuole in a macrophage. Bar is 1 μm. (**F**) Low power of a macrophage with a loose parasitophorous vacuole containing two parasites (enclosed area). HN – host cell nucleus. Bar is 1 μm. (**G**) Detail of the enclosed area in F showing two tachyzoite-like parasites within a PV containing a tubular network. D – dense granule; R – rhoptry. Bar is 1 μm. (**H**) Enlargement of the enclosed area in G showing the tubular structures (T) within the membrane bound PV. Note the strands of host cell endoplasmic reticulum (ER) closely associate with the PVM. Bar is 100 nm.

Co-cultures were fixed and processed for electron microscopy after 24 h of oocyst/macrophage interaction. A major technical problem was that oocysts and sporocysts with intact walls are refractive to fixation and processing for electron microscopy resulting in either the oocyst falling out of the section or undergoing extensive shrinkage (Supplementary Fig. S2A)^41^. Given these limitations it was possible to identify macrophages with one or more (often 3) phagocytized intact oocysts (Supplementary Fig. S2C). At 24 h, the majority of oocysts were fully phagocytized but a few still were attached to macrophage surface with cytoplasmic process partially enclosing the oocyst wall (Supplementary Fig. S2A,B). However, in some cases it was possible to identify well-preserved oocysts and sporocysts with intact sporozoites in the cytoplasm of macrophages (Fig. 6A–C). In these cases, there was evidence of the rupture of the oocyst wall and separation of the plates of the sporocyst wall (Fig. 6B). The separation of the plates of the sporocyst wall appeared to occur at the sutures (Fig. 6D) and was similar to that observed during chemically-induced excystation using digestive components^16^,^17^. In other macrophages, it was possible to identify the remnants of ‘excysted’ oocysts with empty sporocysts lacking sporozoites (Fig. 6D). Although very rare, it was possible to identify sporozoites in the macrophage cytoplasm in cells with oocyst remnants but also in those without such structures (Fig. 6E). These organisms were initially observed in tightly fitting vacuoles but appeared to undergo conversion to give rise to parasitophorous vacuoles (PV) containing tachyzoite-like organisms (Fig. 6F,G). These vacuoles had the characteristic features associated with tachyzoite development^42^,^43^, i.e. having an extensive intra-vacuolar tubular array with host cell endoplasmic reticulum (ER) located adjacent to the PV membrane (Fig. 6F,G). The ultrastructural observation was consistent with the excystation of low numbers of oocysts resulting in the release of sporozoites, which can invade new macrophages giving rise to tachyzoites. The observation of low numbers of tachyzoite-containing vacuoles was consistent with the immune-cytochemical identification of SAG1-positive tachyzoites (Fig. 5).

## Concluding Remarks

The present study demonstrates that mouse macrophage-like RAW cells are able to facilitate the excystation of *T. gondii* sporozoites following internalisation of oocysts. It has been reported previously that infections can result from injection of oocysts to extra-intestinal sites for both *T. gondii* and *Eimeria* spp.^19^,^20^,^37^. In the case of the *Eimeria* species, the sporozoites are able to travel to the intestine and undergo normal coccidian development within the intestinal epithelium. However in these previous studies, the mechanism of excystation and the role of host inflammatory cells were not examined in detail. The present study is, to our knowledge, the first to implicate host naïve macrophages as possible facilitator of the process excystation. Macrophages can tightly adhere oocysts within minutes irrespective of the oocyst surface structure, and engulf them by remodelling their actin cytoskeleton. Opening of the oocyst and sporocyst walls appears to occur within acidic compartments (phagolysosomes) but it is not yet determined which chemical factors are the key players for wall disruption. From the present study, it is evident that some sporozoites are able to escape from phagolysosomes and overcome destruction, perhaps in a similar way as phagocytized tachyzoites^24^. Contribution of phagocytic cells in opening the robust *T. gondii* oocyst *in vitro* is quite unexpected but this could occur following oocyst inoculation at extra-intestinal sites, such as skin, in which macrophages and other inflammatory cells will home on deposits of foreign^44^. It is rather unlikely that macrophages can play a role after ingestion of food or water contaminated with oocysts because phagocytic cells are almost absent of the intestinal epithelium and lumen, and mucus would represent a physical barrier to oocyst attachment to the intestinal cells^45^. It has been suggested that inhalation of oocysts present in dust or aerosol with subsequent swallowing may have been involved in an outbreak of human infection^46^. However, from our *in vitro* observations, it could be proposed that in rare cases, a few oocysts entering the lungs may be exposed to alveolar macrophages possibly resulting in excystation and subsequent infection. Testing this hypothesis is difficult and beyond the scope of the present *in vitro* study. Further studies are required to investigate the role of phagocytic cells in processing *T. gondii* oocysts at extra-intestinal sites of infection.

In conclusion, this study opens new perspectives to identify chemical factors, which could be found both in the macrophage and the intestine, in promoting sporozoite excystation under physiological conditions and proposes an entry path for the reported efficient infection resulting from parenteral inoculation of oocysts.

## Methods

### Cell culture

Mouse macrophage-like cell line RAW 264.7 was purchased from European Collection of Cell Cultures (ECACC, Salisbury, United-Kingdom) and maintained at 37 °C and 6% CO_2_ in plastic 75-cm^2^ flasks containing RPMI 1640 medium (Life Technologies, Saint-Aubin, France) supplemented with 10% heat-inactivated foetal bovine serum (FBS) (Life Technologies), 2 mM L-glutamine, 100 U/ml penicillin and 100 μg/ml streptomycin (Life Technologies). Cells were subcultured twice per week. For this, cells were detached with a cell scraper and ten-fold diluted in fresh medium. Human foreskin fibroblasts (HFF) were obtained from American Type Culture Collection (ATCC, Manassas, USA) and maintained as 80% confluent at 37 °C and 6% CO_2_ in plastic 75-cm^2^ flasks containing RPMI 1640 medium supplemented with 10% heat-inactivated FBS, 2 mM L-glutamine, 100 U/ml penicillin and 100 μg/ml streptomycin.

### *T. gondii* oocysts

Oocysts of the genotype II Me49 strain of *T. gondii*^47^ were harvested from faeces of cat 6–8 days after feeding infected mouse tissues to a *T. gondii* free cat as described previously^8^. This procedure was carried out in accordance with relevant guidelines and regulations following a protocol approved by the Beltsville Area Animal Care and Use Committee (BAACUC), United States Department of Agriculture, Beltsville, MD, USA. Briefly, oocysts were collected by flotation at 4 °C from cat faeces on a 1.15 specific gravity sucrose solution, washed in distilled water and then resuspended in 7 ml of an aqueous solution containing 2% H_2_SO_4_. Oocysts were allowed to sporulate at room temperature (RT 20–22 °C) for 7 days under adequate aeration and gentle continuous shaking. Oocysts were then sent with cold packs within 48 h from Beltsville, Maryland, USA to Marseille, France, for further experiments. Upon reception, oocyst suspension was stored in a 2% H_2_SO_4_ aqueous solution at 4 °C until used^8^. This suspension was referred to contain ‘native’ oocysts.

### Time-lapse microscopy

Macrophages were cultivated in RPMI medium in glass bottom Petri dishes (FluoroDish, WPI Instruments) at 37 °C, 6% CO_2_ in a cell incubator for a 24-h period. The day of the experiment, the Petri dish containing macrophages was placed on a microscope (Zeiss Axiovert 200, equipped with 10x, 20x, 40x lenses and an CoolSnap HQ2 camera) equipped with a chamber heated at 37 °C (JPK PetriDish Heater, JPK Instrument)^48^. A small sample of oocysts was then added in the Petri dish above the macrophages to reach approximately a 1:1 ratio. The co-cultures were filmed at 20x magnification in transmission for a 1 to 2-h period with a frame rate of 1 per 2 minutes using μManager (https://micro-manager.org/) software. All movies and images were analyzed using Fiji/ImageJ software (http://fiji.sc/Fiji).

### Micropipette aspiration

A glass capillary (ID 0.58 mm/OD 1 mm; Sutter Instruments Ref. B100-58-10) was heated using a micropipette puller (David Kopf Instruments, Model 700C) until melting and separated in half to obtain two closed microneedles. Then, the tips of the two microneedles were forged as micropipettes under microscope (Microforge, Alcatel) until the desired shape and size of the opening was obtained (typically 5 μm). Both micropipettes were filled with PBS/1% BSA medium carefully to passivate their inner walls. The observation chamber (made of two 22 mm × 10 mm cuts of 170 μm-thick coverslide separated by ~1 mm using a homemade holder and vacuum grease) was, prior to oocyst and macrophage introduction, incubated with PBS/1% BSA to ensure that the cells do not adhere to the bottom slide to ease cell and parasite captures. The pipettes were introduced in the chamber to passivate their outer wall. They were mounted on x, y, z micromanipulator (one Narishige hydrolic system, one Sutter Instruments MP285 electronic one), facing each other on an inverted microscope (Olympus IMT2, equipped with a Prosilica GE680 camera) at x40 magnification. Aspiration of cells in suspension was performed after exchange of the buffer with RPMI, introduction of diluted cells and parasites and their sedimentation. using a homemade aspiration system based on interconnected vessels and the aspiration pressure was monitored using pressure sensors. After exchange of the buffer with RPMI, introduction of diluted cells and parasites and their sedimentation, the sequence of micromanipulation events was set as follow. A non-adherent macrophage and one oocyst were respectively selected and aspirated with each micropipette at controlled pressures (typically 1–3 cm H_2_O for the macrophage, and >5 cm H_2_O for the oocyst). The oocyst (which was observed not to deform at all) was gently presented and pressed to the macrophage for ~60 seconds and aspiration was then quickly released on the oocyst, and the holding pipette removed while the macrophage/oocyst pair was observed over time for at least 10 min. Images were acquired using a homemade software programmed in Labview (http://www.ni.com/labview/) and analyzed using Fiji software. The micropipettes and oocyst/macrophage suspensions were changed every hour. Experiments were run at room temperature.

### Bleach treatment of the oocysts

In order to determine if removal of the outer oocyst wall layer restricts or enhances oocyst interactions with RAW macrophages, oocysts were exposed to bleach solution as described previously^8^. For this, oocysts were washed three times in PBS at 10,000 × g for 2 min to remove sulphuric acid traces. Bleach treatment consisted in incubating oocysts with 1 ml of a diluted household bleach solution (Javel Lacroix, Colgate-Palmolive company, Bois-Colombes, France) containing 3% sodium hypochlorite in PBS for 30 min at 4 °C. Oocysts were then washed three times in PBS at 10,000 × g for 2 min and kept at 4 °C until used.

### Internalisation assay

Confluent RAW 264.7 macrophages were detached with a cell scraper and plated in triplicate overnight at 37 °C and 6% CO_2_ onto 8-well Permanox Lab-Tek chamber slides (Dutscher, Brumath, France) at the density of 1.5 × 10^5^ cells per well. Then, 1.5 × 10^5^ oocysts, either bleach-treated (as described above) or not, were added in each well and incubated with macrophages for 1, 4 and 24 h in culture conditions. After that, preparations were fixed in 4% (w/v) paraformaldehyde (PFA) in PBS 20 min at 4 °C, rinsed three times with PBS, and then permeabilized with 0.1% TritonX-100 in PBS for 15 min at RT. Macrophages were stained by rhodamine-conjugated phalloidin (Life Technologies) diluted at 1:40 in PBS for observation of F-actin filaments. After several washes with PBS, slides were air-dried and mounted with slow-fast DAPI medium (Life Technologies). Bright field and epifluorescence images of fixed cells were collected on an Olympus BX 51 equipped with a cooled CCD camera (XC30, Olympus), 40× and 100× objectives (Olympus), and suitable epifluorescence filters for DAPI, FITC and rhodamine. Images were acquired using the fluorescence imaging system CellA (Olympus, France) and were further analyzed using ImageJ 1.49d (http://imagej.nih.gov/ij/).

The following parameters were considered in order to describe the kinetics of parasite internalisation over a 24 h period: the number of oocyst substructures (i.e. either full oocysts or free sporocysts) that were attached to or internalised by macrophages, and the number of macrophage cells containing oocyst substructures were counted manually over randomly selected 20–40 fields (~150–200 macrophages) per well and condition. The percentage of attached vs. internalised oocysts/sporocysts, the percentage of macrophages containing oocysts/sporocysts, and the number of oocysts/sporocysts per macrophage were calculated after 1, 4 or 24 h incubation time.

### Inhibition of the oocyst internalisation

Macrophages were pre-incubated at 37 °C for 1 h with 10 μM of the F-actin polymerisation inhibitor cytochalasin D (CytD, Sigma-Aldrich, Saint-Quentin-Fallavier, France) or 100 nM of the phosphoinositide 3-kinase (PI3K) inhibitor wortmannin (WT, Sigma-Aldrich). The latter impairs the closure of phagosomes but not the phagocytic cup formation^31^,^32^.

Then, native oocysts were added at ratio 1:1 and incubated with macrophages at 37 °C for 1 h in continuous presence of CytD or WT. Cells incubated with 0.1% DMSO served as carrier control. After fixation and staining of F-actin filaments by rhodamine-conjugated phalloidin, the percentage of internalising macrophages and the phagocytic index (PI) were calculated. PI was defined as the mean over the different repetitions of the total number of internalised oocysts divided by the total number of macrophage cells, i.e. either hosting an oocyst or not^49^.

### Tracking of acidic compartments in RAW macrophages

Briefly, macrophages were plated overnight onto 8-well Permanox Lab-Tek chamber slides (Dutscher, Brumath, France) chamber slide at the density of 1.5 × 10^5^ cells per well as described above. RPMI medium was then discarded and replaced with a solution of 100 nM red LysoTracker^®^ DND-99 (Life Technologies) in RPMI medium. Cells were incubated for 2 h at 37 °C, 6% CO_2_. Then, oocysts were added at 1:1 ratio and let in co-incubation for 24 h at 37 °C, 6% CO_2_. Macrophages incubated in the absence of oocysts served as negative controls. After medium removal, rinsing and fixation, cells were observed under bright field and epifluorescence for LysoTracker red fluorescence (530–550 nm/590 nm), DAPI and oocyst natural fluorescence. Images were analyzed by ImageJ 1.49 as described above.

The percentage of LysoTracker positive macrophages was determined by manual counting over about 800 cells per experiment. Among them, we carefully categorized (i) macrophages harbouring LysoTracker-positive granules located around ingested oocysts, (ii) macrophages containing at least one LysoTracker-positive compartment colocalizing with at least one ingested oocyst, and (iii) macrophages that did not apparently ingested any oocyst but harboured LysoTracker positive granules.

### Monitoring the development of tachyzoites in oocyst-macrophage co-cultures

RAW macrophages were plated overnight at the density of 5.10^4^ cells/well and then co-incubated with native oocysts at ratio 1:1 for 24 h as described above. HFF cells incubated with oocysts served as controls for possible sporozoite excystation in absence of phagocytosis by RAW cells, either by direct interaction with HFF cells or following exposure at 37 °C for 24 h. After incubation, RAW and HFF cells were fixed in absolute cold methanol for 10 min and rinsed three times with PBS. Cells were incubated with a rabbit polyclonal tachyzoite-specific anti-SAG1 antibody^50^ at 1:1000 dilution in PBS and then with a corresponding FITC-conjugated secondary antibody. Slides were then processed as described above for detection of SAG1-positive tachyzoites by fluorescence microscopy. Parasitophorous vacuoles containing either a single, two or more than two tachyzoites were manually counted in each well.

### Monitoring the development of tachyzoites in macrophage cultures challenged with bile-excysted sporozoites

To obtain sporozoites, ~10^7^ oocysts in 300 μl PBS were sonicated in a Branson 200 water bath in a plastic 1.5-ml microtube for 10 min at RT. Under these conditions, sonication results in >95% free intact sporocysts. Sporocysts were then pelleted by centrifugation at 10,000 × g for 2 min and resuspended in 1 ml of excystation medium containing 0.75% (w/v) bile extract (Sigma-Aldrich, #S9875) and 0.1% sodium bicarbonate in Hanks’ balanced salt solution. After incubation for 2 h at 37 °C, excysted sporozoites were washed twice in 1 ml RPMI1640 + 10% FBS culture medium by centrifugation at 5,000 × g for 5 min and then suspended in culture medium. Freshly excysted sporozoites were used immediately for macrophage invasion assay. For this, macrophages were plated the day before and incubated overnight in triplicate at the density of 1.5.10^5^ cells on 12 mm-glass coverslips precoated with 0.01% poly-L-lysine solution and then co-incubated with a quantity of sporozoites equivalent to 1.5.10^5^ oocystes (i.e. ~1.2.10^6^ sporozoites assuming that one fully sporulated oocyst contained eight sporozoites) or with native intact oocysts at ratio 1:1 for 24 h. After incubation, RAW cells were fixed in absolute cold methanol for 10 min and rinsed three times with PBS. Cells were then incubated at 37 °C for 1 h with a human antiserum reacting with both *T. gondii* sporozoites and tachyzoïtes at 1:200 dilution in PBS and a rabbit polyclonal sporozoite-specific anti-AMA4 antibody^51^ at 1:1000 dilution in PBS. After three washes in PBS, cells were then incubated at 37 °C for 1 h with a FITC-conjugated secondary anti-human antibody at 1:100 dilution in PBS and an Alexa546-conjugated secondary anti-rabbit antibody at 1:50 dilution in PBS. Slides were then processed as described above for detection of sporozoites and tachyzoites by fluorescence microscopy. The percent of macrophages containing sporozoites or tachyzoites was determined by manually counting ~200 cells per coverslip and condition.

### Transmission electron microscopy (TEM) on oocyst-macrophage co-cultures

Macrophage cells were cultured for 3 days in 75-cm^2^ flasks prior to be incubated for 24 h with native oocysts at ratio 1:1. Then, cells were washed twice with 0.1 M phosphate buffer and gently detached with a cell scrapper. Following centrifugation (400 g, 10 min), cells were resuspended and fixed in 2.5% glutaraldehyde in 0.1 M phosphate buffer. The samples were post-fixed in osmium tetroxide, dehydrated in ethanol, treated with propylene oxide and embedded in Spurr’s epoxy resin. Thin sections were cut and stained with uranyl acetate and lead citrate prior to examination in a Jeol 1200EX electron microscope.

### Statistical analysis

All data were analyzed by using GraphPad Prism 5.0 software. Pooled data were transformed to log10 values and were found to follow a normal distribution by using the d’Agostino & Pearson omnibus normality test. Statistical significance between the data sets was evaluated by one-way ANOVA tests followed by the Bonferroni’s multiple comparison test. p value < 0.05 was considered significant: ***, **, and * indicates p < 0.001, p < 0.01, and p < 0.05 respectively. Unless stated, data were expressed as the mean ± standard deviation of four independent experiments.

## Additional Information

**How to cite this article**: Freppel, W. *et al.* Macrophages facilitate the excystation and differentiation of *Toxoplasma gondii* sporozoites into tachyzoites following oocyst internalisation. *Sci. Rep.* **6**, 33654; doi: 10.1038/srep33654 (2016).

## Supplementary Material

Supplementary Information

Supplementary MOvie S1

Supplementary MOvie S2

Supplementary MOvie S3

Supplementary MOvie S4
