# Supplementary material for: Macrophages facilitate the excystation and differentiation of Toxoplasma gondii sporozoites into tachyzoites following oocyst internalisation
Source: Sci Rep. 2016 Sep 19;6:33654. doi: 10.1038/srep33654 (PMC5027544; doi:10.1038/srep33654)
Supplement: Supplementary Information [file srep33654-s1.pdf]

SUPPLEMENTARY INFORMATION

Macrophages facilitate the excystation and differentiation of *Toxoplasma gondii* sporozoites into tachyzoites following oocyst internalisation

Wesley Freppel[1], Pierre-Henri Puech[2,3,4], David J. P. Ferguson[5], Nadine Azas[1], Jitender P. Dubey[6], Aurélien Dumètre[1,*]

[1] Aix Marseille Université, IP-TPT UMR MD 3, Marseille, F-13385, France.
[2] Aix Marseille Université, LAI UM 61, Marseille, F-13288, France.
[3] Inserm, UMR_S 1067, Marseille, F-13288, France.
[4] CNRS, UMR 7333, Marseille, F-13288, France.
[5] Nuffield Department of Clinical Laboratory Science, University of Oxford, John Radcliffe Hospital, Oxford, OX3 9DU, United Kingdom.
[6] United States Department of Agriculture, Agricultural Research Service, Beltsville Agricultural research Center, Animal Parasitic Diseases Laboratory, Building 1001, Beltsville, MD 20705-2350, USA.

*Corresponding author: Aurélien Dumètre; Email: aurelien.dumetre@univ-amu.fr; Tel: +33 491-835-544

Figure S1: Development of *T. gondii* tachyzoites in RAW macrophages challenged with oocysts or bile-excysted sporozoites. Macrophage cells were incubated with oocysts (ratio 1:1) or bile-excysted sporozoites (ratio 1:8) for 24 h at 37°C, then fixed and stained for macrophage nucleus (blue), sporozoite/tachyzoite (green), and sporozoite-specific (red) identification. (A) Percent of macrophages containing either excysted sporozoites or tachyzoites following incubation with oocysts or bile-excysted sporozoites. **, p<0.01; ***, p<0.001. (B) Very few sporozoites (arrowheads) were still detected at 24 hr. Sporozoites were always seen contained within internalised oocysts in macrophage-oocyst co-cultures (1st line) and free in macrophage cells incubated with bile-excysted sporozoites, sometimes alongside tachyzoites (arrows) in the same cell (2nd line). Scale bars: 10 µm.

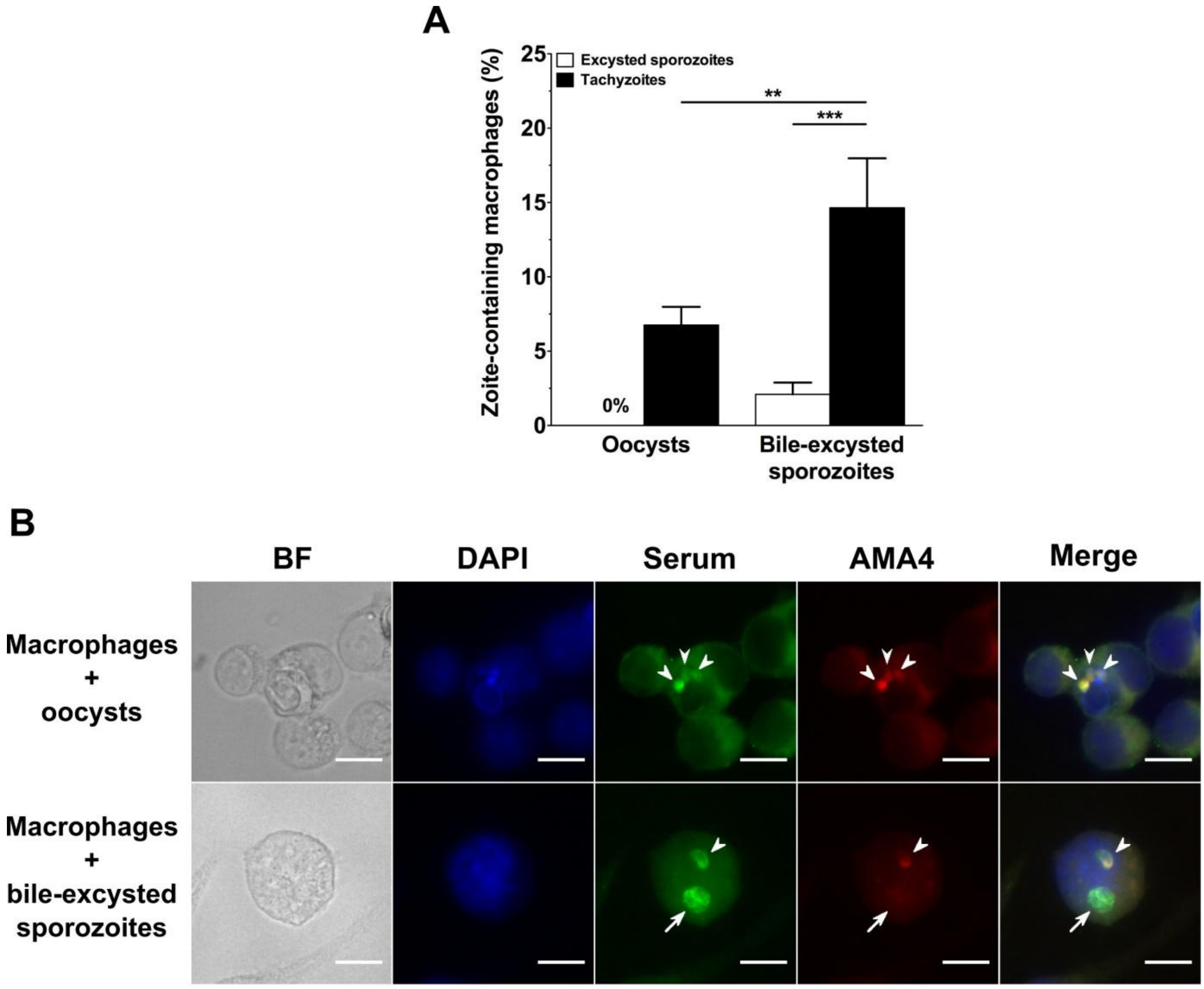

Figure S2: Ultrastructural appearance of oocyst – macrophage co-cultures. (A) Section through a macrophage showing a poorly preserved internalised oocyst and one adhering to the surface. N - macrophage nucleus; Oo - oocyst. Bar is 1 µm. (B) Detail of the enclosed area in A showing pseudopodia from the macrophage partially enclosing the surface of the oocyst (arrowheads). Bar is 1 µm. (C) Section through a macrophage containing three shrunken oocysts (Oo). N – macrophage nucleus. Bar is 1 µm.

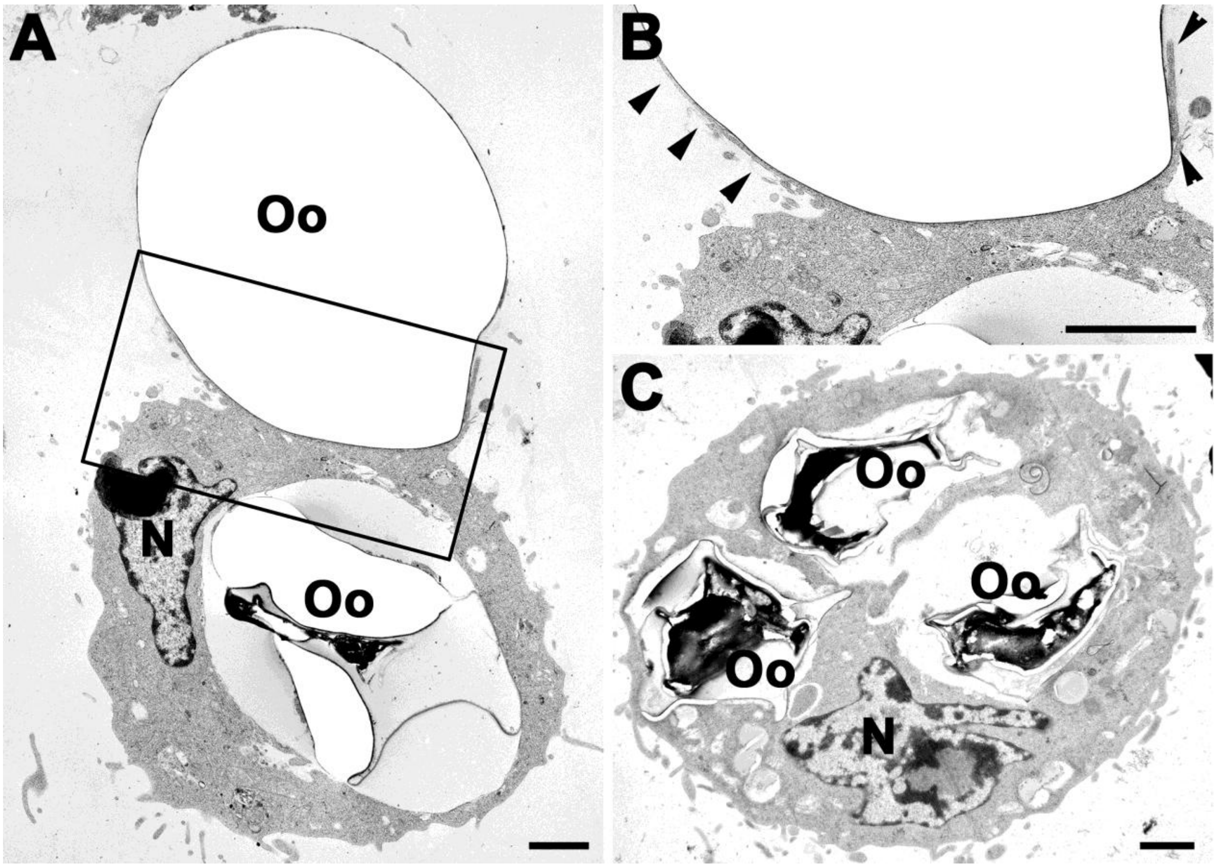

Movie S1: Time-lapse video microscopy of a RAW macrophage cell catching *T. gondii* oocysts. Note that the macrophage cell clearly moves towards the first oocyst before managing two additional oocysts that subsequently appear within the same focal plane. The movie starts at t=16 min, i.e. 16 min after introducing oocysts in the macrophage cell culture. At this time, most of oocysts located in the same focal plane as the macrophage cells. Time interval = 2 min. Scale bar = 10µm. Movie runs at 2 frames per second.

Movie S2: Time-lapse video microscopy of two RAW macrophage cells engulfing each a *T. gondii* oocyst. These oocysts are labeled with yellow arrowhead until the macrophage cells internalise them. The movie starts at t=16 min, i.e. 16 min after introducing oocysts in the macrophage cell culture. At this time, most of oocysts located in the same focal plane as the macrophage cells. Time interval = 2 min. Scale bar = 10µm. Movie runs at 2 frames per second.

Movie S3: Time-lapse video microscopy of a RAW macrophage cell engulfing two *T. gondii* oocysts. These oocysts are labeled with yellow arrowhead until the macrophage cells internalise them. Note that two supplementary oocysts remain attached to the cell surface during all the observation time. The movie starts at t=16 min, i.e. 16 min after introducing oocysts in the macrophage cell culture. At this time, most of oocysts located in the same focal plane as the macrophage cells. Time interval = 2 min. Scale bar = 10µm. Movie runs at 2 frames per second.

Movie S4: Time-lapse video microscopy of two RAW macrophage cells competing for a *T. gondii* oocyst. Note the apparent stretching of the cytoplasm of one of the two macrophage cells holding the oocyst from t=118 min (arrow). The movie starts at t=24 min, i.e. 24 min

after introducing oocysts in the macrophage cell culture. At this time, most of oocysts located in the same focal plane as the macrophage cells. Time interval = 2 min. Scale bar = 10µm. Movie runs at 2 frames per second.
